# Supplementary material for: Oncogenic PIK3CA corrupts growth factor signaling specificity
Source: Mol Syst Biol. 2024 Dec 20;21(2):126–57. doi: 10.1038/s44320-024-00078-x (PMC11791070; doi:10.1038/s44320-024-00078-x)
Supplement: Supplementary file 1 — Appendix [file 44320_2024_78_MOESM1_ESM.pdf]

Oncogenic *PIK3CA* corrupts growth factor signaling specificity

Authors: Ralitsa R. Madsen[1,2,*], Alix Le Marois[3,†], Oliwia N. Mruk[2,†], Margaritis Voliotis[4,†], Shaozhen Yin[1,†], Jahangir Sufi[5], Xiao Qin[5,8], Salome J. Zhao[1], Julia Gorczynska[1], Daniele Morelli [1], Lindsay Davidson[6], Erik Sahai[3], Viktor I. Korolchuk[7], Christopher J. Tape[5], Bart Vanhaesebroeck[1]

Affiliations:

[1]Cell Signaling Laboratory, Department of Oncology, University College London Cancer Institute Paul O'Gorman Building, University College London; London, WC1E 6BT, UK.

[2]MRC Protein Phosphorylation and Ubiquitylation Unit, School of Life Sciences, University of Dundee; Dundee, DD1 5EH, UK.

[3]Tumour Cell Biology Laboratory, The Francis Crick Institute; London, NW1 1AT, UK.

[4]Department of Mathematics and Statistics and Living Systems Institute; University of Exeter, Exeter, EX4 4QD, UK.

[5]Cell Communication Lab, Department of Oncology, University College London Cancer Institute; London, WC1E 6BT, UK.

[6]Human Pluripotent Stem Cell Facility, School of Life Sciences, University of Dundee; Dundee, DD1 5EH, UK.

[7]Biosciences Institute, Faculty of Medical Sciences, Newcastle University; Newcastle upon Tyne, NE4 5PL, UK.

[8]Current: MRC Translational Immune Discovery Unit, MRC Weatherall Institute of Molecular Medicine, Oxford, OX3 9DS, UK.

[†]Contributed equally

[#]Corresponding author. Email: rmadsen001@dundee.ac.uk

Table of Contents

**Content**	**Title**	**Page**
Appendix Figure S1	CRISPR/Cas9 *PIK3CA* exon 21 engineering and quality control assays of HeLa clones	3
Appendix Fgure S2	Light microscopy images of HeLa and iPSC maintenance cultures with different *PIK3CA* genotypes	5
Appendix Table S1	Primary antibodies for Western blotting	6
Appendix Table S2	Secondary antibodies for Western blotting	7
Appendix Table S3	Mass cytometry (CyTOF) antibodies	7
Appendix Table S4	RT-qPCR primers	8

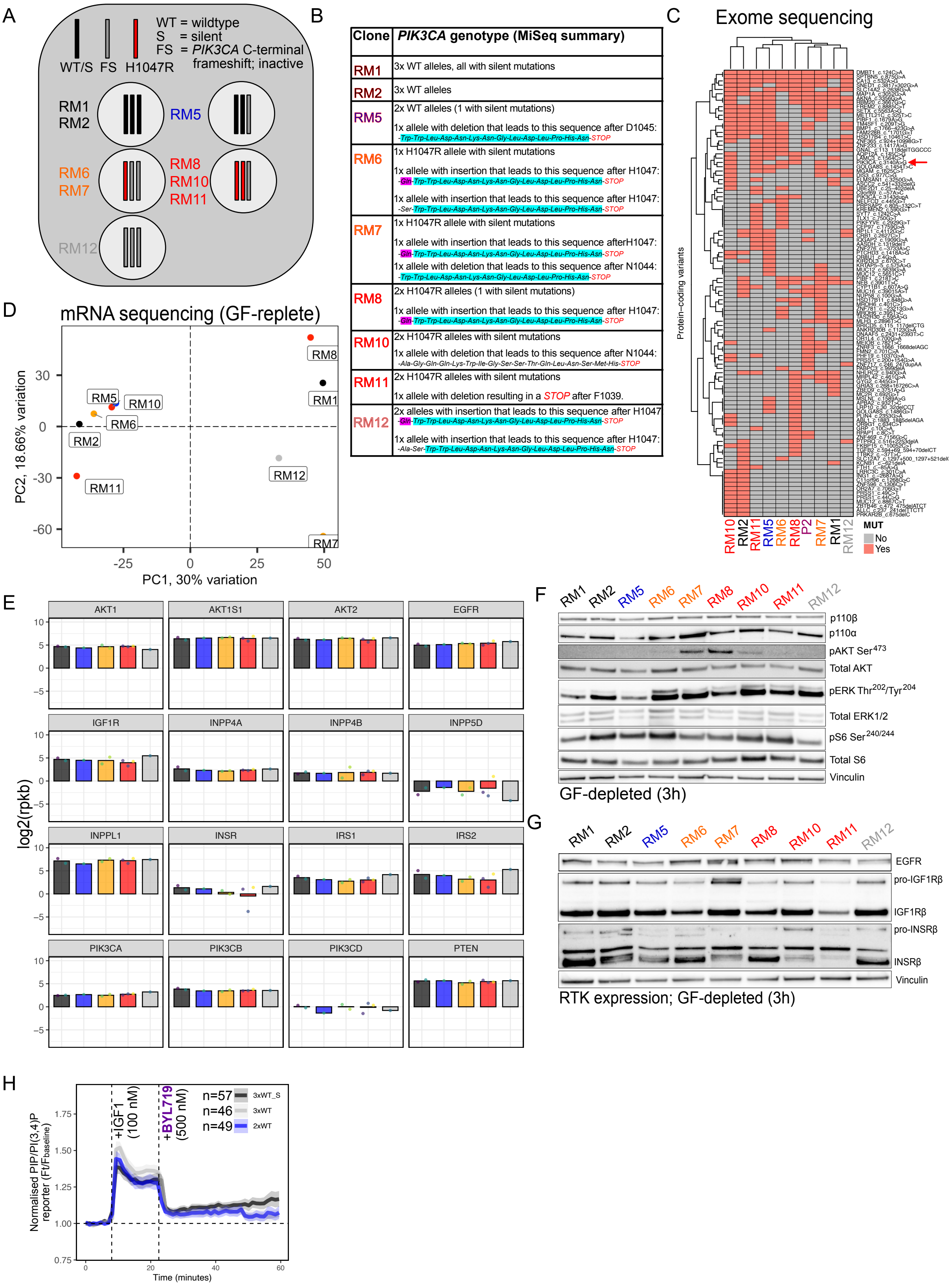

Appendix Figure S1. CRISPR/Cas9 *PIK3CA* exon 21 engineering and quality control assays of HeLa clones. (A) Summary of the final set of clones that were banked and validated following engineering. Because HeLa cells are nominally triploid, the edited clones either have three WT *PIK3CA* alleles or either one of the following: 0-2 WT *PIK3CA* alleles, 0-2

PIK3CA^{H1047R} alleles and/or 0-3 C-terminal frameshift alleles. **(B)** The C-terminal frameshift truncation results in recoding of the C-terminal 20-30 amino acids of the p110α protein (common reading frames are highlighted in purple and turquoise). This abolishes the critical WIF motif in p110α required for membrane binding and catalytic function (Jenkins et al. 2023 doi: 10.1038/s41467-023-35789-6), effectively creating a loss-of-function knock-in that does not carry the risk of a complete knock-out in terms of altering the stoichiometry of regulatory and catalytic p110 subunits. The exact allelic sequence was obtained following targeted next generation sequencing of the edited *PIK3CA* exon 21 region. **(C)** Clustering of individual HeLa cultures based on protein-coding gene variants found in two or more CRISPR/Cas9 engineered HeLa clones relative to the parental culture prior to gene editing. Gene variants were identified using whole exome sequencing, which included a control HeLa culture passaged alongside the CRISPR/Cas9-edited clones without any editing or subcloning. A red arrow is used to indicate the *PIK3CA*^{H1047R} edit which is the only protein-coding variant common to all *PIK3CA*^{H1047R} mutant cell lines. **(D)** Principal component analysis (PCA) of CRISPR/Cas9-engineered HeLa clones based on total mRNA sequencing data obtained in baseline culture condition following fresh medium replenishment 3 h prior to sample collection. The observed clustering is similar to that observed with the exome sequencing data in (C), without any systematic differences driven by the presence of the *PIK3CA*^{H1047R} variant. **(E)** Barplots comparing the expression levels in log2(reads per kilobase) for selected genes encoding PI3K pathway-relevant components and receptor tyrosine kinases. **(F)** Western blots for PI3K signaling components and pERK1/2 following 3 h of serum or growth factor (GF) removal, using all CRISPR/Cas9-engineered HeLa clones. **(G)** Western blots for relevant receptor tyrosine kinases following 3 h of serum or growth factor (GF) removal, using all CRISPR/Cas9-engineered HeLa clones. EGFR, epidermal growth factor receptor; IGF1R, insulin-like growth factor 1 receptor; INSRβ, insulin receptor β chain. **(H)** TIRF-M of the PH-AKT2-derived biosensor in HeLa clones RM1 (3xWT_S), RM2 (3xWT) and RM5 (2xWT) stimulated with 100 nM IGF1, then treated with the PI3Kα inhibitor BYL719 (500 nM). The data are from 2-4 independent experiments per clone. The traces represent mean fold-change relative to baseline (the median signal of the first seven time points), with shaded areas representing bootstrapped 95% confidence intervals of the mean.

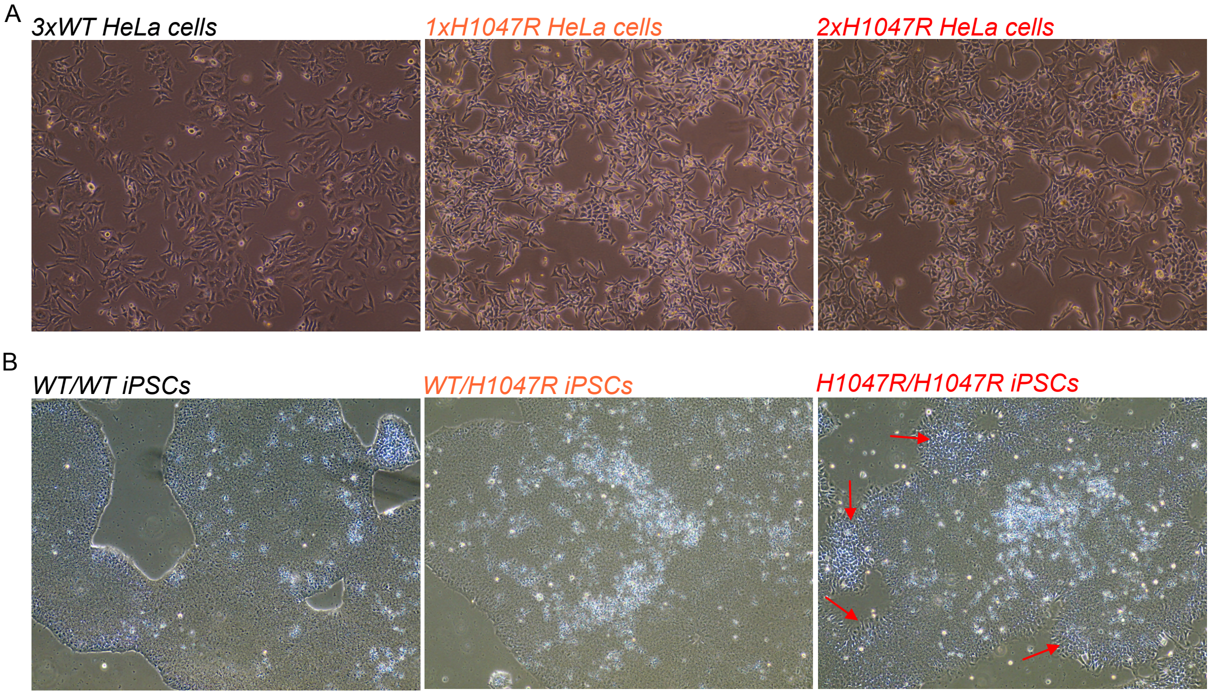

Appendix Figure S2. Light microscopy images of HeLa and iPSC maintenance cultures with different *PIK3CA* genotypes. (**A** and **B**) The HeLa cultures in A correspond to independent CRISPR/Cas9-engineered HeLa clones to those used for staining in main Fig. 5B. The red arrows in (B) point to example regions in iPSC cultures with disorganized colony growth and mesenchymal-like morphology changes in homozygous *PIK3CA*^{H1047R} iPSCs. The images are representative of n=5 wild-type, n=3 heterozygous and n=9 homozygous iPSC clones (see also Madsen et al. 2019 doi: 10.1073/pnas.1821093116).

Antibody target	Clone	Mol. Weight (kDa)	Lot #	Vendor	Cat. # (RRID)	Dilution
IGF1Rβ (C-20)	C-20	95	J1907	Santa Cruz	sc-713 (AB_671792)	1:200
INSRβ (C-20)	C-20	95	L1812	Santa Cruz	sc-711 (AB_631835)	1:200
EGFR	Polyclonal	175	17	CST	2232 (AB_331707)	1:1000
p110α	C73F8	110	11	CST	4249 (AB_2165248)	1:1000
p110β	C33D4	110	8	CST	3011 (AB_2165246)	1:1000
pAKT Thr^{308}	244F9	60	17	CST	4056 (AB_331163)	1:1000
pAKT Ser^{473}	Polyclonal	60	14	CST	9271 (AB_329825)	1:1000
Total AKT	Polyclonal	60	28	CST	9272 (AB_329827)	1:1000
pERK1/2 Thr^{202}; Tyr^{204}	Polyclonal	44/42	17	CST	4370 (AB_2315112)	1:1000
pS6 $Ser^{240/244}$	Polyclonal	32	18	CST	2215 (AB_331682)	1:1000
Total S6	54D2	32	13	CST	2317 (AB_2238583)	1:1000
Vinculin		124	NA	Sigma Aldrich	V9131 (AB_477629)	1:5000

Appendix Table S1: Primary antibodies for Western blotting

Antibody	Vendor	Lot #	Cat. #	Dilution
anti-rabbit HRP-conjugated	Amersham	17203153	NA934V	1:5000
anti-mouse HRP-conjugated	Amersham	17193521	NXA931V	1:5000
Goat anti-rabbit IgG HRP-linked antibody	CST	30	7074S	1:10000
Goat anti-mouse IgG HRP-linked antibody	CST	36	7076S	1:10000

Appendix Table S2: Secondary antibodies for Western blotting

Antibody target	Clone	Metal	Lot #	Vendor	Cat. # (RRID if available)	Amount (1 rxn)
Total S6	54D2	141-Pr	16	CST	2317 (AB_2238583)	0.32 µg
Cleaved caspase 3 Asp^{175}	D3E9	142-Nd	4	CST	9579 (AB_10897512)	0.59 µg
pRB $Ser^{807/811}$	J112-906	150-Nd	2005420	Fluidigm	3150013	0.7 µg
pNDRG1 Thr^{346}	D98G11	151-Eu	4	CST	5482 (AB_10693451)	0.56 µg
pAKT Ser^{473}	M89-61	155-Gd	20	BD Biosciences	560397 (AB_1645553)	4 µg
pERK1/2 Thr^{202}; Tyr^{204}	20A	167-Er	1263406	BD Biosciences	612359 (AB_399648)	0.09 µg
pSMAD2/3 $Ser^{465/423}$ $Ser^{467/425}$	D27F4	168-Er	2	CST	8828 (AB_2631089)	1.72 µg
pS6 $Ser^{240/244}$	D68F8	173-Yb	7	CST	5364 (AB_10694233)	0.46 µg

Appendix Table S3: Mass cytometry (CyTOF) antibodies

**Gene target**	**Forward primer (5'-3')**	**Reverse primer (5'-3')**
TBP	TAATCCCAAGCGGTTTGC	TAGCTGGAAAACCCAACTTCT
SNAI2 (Slug)	GCCTCCAAAAAGCCAAACTACA	GAGGATCTCTGGTTGTGGTATGACA
SNAI1 (Snail)	TTCTCACTGCCATGGAATTCC	GCAGAGGACACAGAACCAGAAA
ACTB	CGGGAAATCGTGCGTGACATTAAG	TGATCTCCTTCTGCATCCTGTCGG
c-FOS	GGGGCAAGGTGGAACAGTTAT	AGGTTGGCAATCTCGGTCTG
c-JUN	TCCAAGTGCCGAAAAAGGAAG	CGAGTTCTGAGCTTTCAAGGT
EGR1	CACCTGACCGCAGAGTCTTTT	CAGGGAAAAGCGGCCAGTAT
FOSL1	AAGGCCTTGTGAACAGATCAGC	GTCAGTTCCTTCCTCCGGTT
JUNB	AAGGGACACGCCTTCTGAAC	AAACGTCGAGGTGGAAGGAC

Appendix Table S4: RT-qPCR primers
