# Supplementary material for: Oncogenic PIK3CA corrupts growth factor signaling specificity
Source: Mol Syst Biol. 2024 Dec 20;21(2):126–57. doi: 10.1038/s44320-024-00078-x (PMC11791070; doi:10.1038/s44320-024-00078-x)
Supplement: Supplementary file 3 — Expanded View Figures [file 44320_2024_78_MOESM3_ESM.pdf]

Expanded View Figures

Figure EV1. Systematic benchmarking of pleckstrin homology (PH) domain-based class I PI3K biosensors.

(**A**) Schematic of the optimized live-cell imaging setup to ensure that multiple comparisons could be performed in the same microenvironment, aided by fluidics for minimal physical perturbation during compound additions. To bring down the baseline of PI3K signaling, serum was removed from the cells 3 h prior to imaging start. D1, D2, D3 refer to day 1, day 2 and day 3 of the experimental workflow. (**B**) Schematic of the different wild-type and mutant PH domain constructs used for benchmarking, all cloned into the same plasmid backbone for consistent comparisons. The portion of the PIP_3-binding region in the PH domain of GRP1, which has often been used for live-cell detection of PIP_3, has an identical sequence to that in the ARF GEF ARNO. We therefore chose to include the latter in our comparisons given the tandem-dimer, modified version of this PH domain as biosensor for PIP_3 (Goulden et al, 2019).The shown alignments cover the conserved β1 strand, variable loop 1, and β2 strand of the PH domain fold. Of the four PH domains, only PH-AKT2 is capable of binding both PIP_3 and $PI(3,4)P_2$. The remaining PH domains only bind PIP_3 (Posor Y et al, 2022). The alanine (A) mutation in the phosphoinositide (PI) signature motif renders the mCherry-tagged mutant PH domain versions unable to bind phosphoinositides. (**C**) Quantification of total internal reflection fluorescence (TIRF) microscopy experiments comparing the response rate and dynamic range of individual PH domain-based PI3K reporters in response to pharmacological PI3Kα activation in HeLa cells. To correct for non-specific increases in biosensor signal at the plasma membrane, the intensity of each GFP-tagged wild-type PH domain was normalized to that of its mCherry-tagged mutant version. Experimental replicates and single-cell numbers are indicated. Two different configurations were tested for the BTK-derived PH domain: with and without the adjacent Tec homology (TH) domain. Only one experiment was performed with PH-BTK without TH because most of the cells failed to tolerate its expression. (**D**) TIRF microscopy of the PH-AKT2-derived biosensor in HeLa cells stimulated with 5 μM 1938, then treated with the PI3Kα inhibitor BYL719 (500 nM). Two independent experiments are superimposed to illustrate the expected inter-experimental variability. (**E**) Evaluation of the performance of the PH-TH version of BTK with N-terminal or C-terminal fluorescent protein fusion, with simultaneous removal of the nuclear export sequence, as in the original plasmid DNA used for subcloning of this reporter. All plots in (**C**, **D**, **E**) represent mean normalized reporter signal relative to time 0, with shading corresponding to +/- 1 standard deviation (SD).

 © The Author(s)

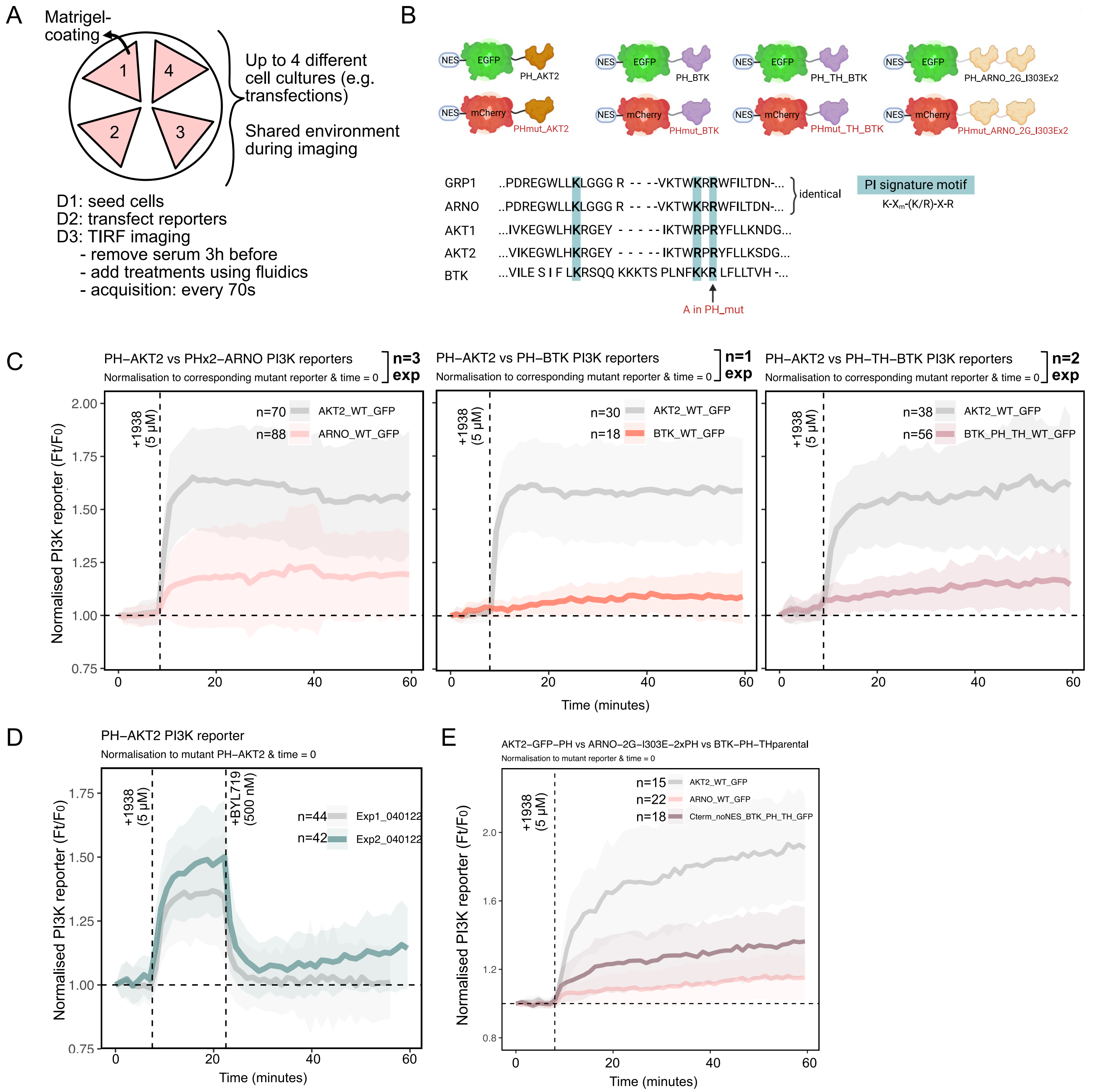
A
Matrigel-coating
1
4
2
3
Up to 4 different cell cultures (e.g. transfections)
Shared environment during imaging
D1: seed cells
D2: transfect reporters
D3: TIRF imaging
- remove serum 3h before
- add treatments using fluidics
- acquisition: every 70s
B
NES EGFP PH_AKT2
NES mCherry PHmut_AKT2
NES EGFP PH_BTK
NES mCherry PHmut_BTK
NES EGFP PH_TH_BTK
NES mCherry PHmut_TH_BTK
NES EGFP PH_ARNO_2G_I303Ex2
NES mCherry PHmut_ARNO_2G_I303Ex2
GRP1 ..PDREGWLLKLGGG R - - - -VKTWKRRWFILTDN-...
ARNO ..PDREGWLLKLGGG R - - - -VKTWKRRWFILTDN-...
identical
AKT1 ...IVKEGWLHKRGEY - - - - - IKTWRPRYFLLKNDG...
AKT2 ...VIKEGWLHKRGEY - - - - - IKTWRPRYFLLKSDG...
BTK ...VILE S I F LKRSQQ KKKTS PLNFKKR LFLLTVH -...
A in PH_mut
PI signature motif
K-Xm-(K/R)-X-R
C
PH–AKT2 vs PHx2–ARNO PI3K reporters
Normalisation to corresponding mutant reporter & time = 0
n=3 exp
PH–AKT2 vs PH–BTK PI3K reporters
Normalisation to corresponding mutant reporter & time = 0
n=1 exp
PH–AKT2 vs PH–TH–BTK PI3K reporters
Normalisation to corresponding mutant reporter & time = 0
n=2 exp
n=70 AKT2_WT_GFP
n=88 ARNO_WT_GFP
n=30 AKT2_WT_GFP
n=18 BTK_WT_GFP
n=38 AKT2_WT_GFP
n=56 BTK_PH_TH_WT_GFP
+1938 (5 μM)
Normalised PI3K reporter (Ft/F0)
Time (minutes)
D
PH–AKT2 PI3K reporter
Normalisation to mutant PH–AKT2 & time = 0
+1938 (5 μM)
+BYL719 (500 nM)
n=44 Exp1_040122
n=42 Exp2_040122
Normalised PI3K reporter (Ft/F0)
Time (minutes)
E
AKT2–GFP–PH vs ARNO–2G–I303E–2xPH vs BTK–PH–THparental
Normalisation to mutant reporter & time = 0
n=15 AKT2_WT_GFP
n=22 ARNO_WT_GFP
n=18 Cterm_noNES_BTK_PH_TH_GFP
+1938 (5 μM)
Normalised PI3K reporter (Ft/F0)
Time (minutes)

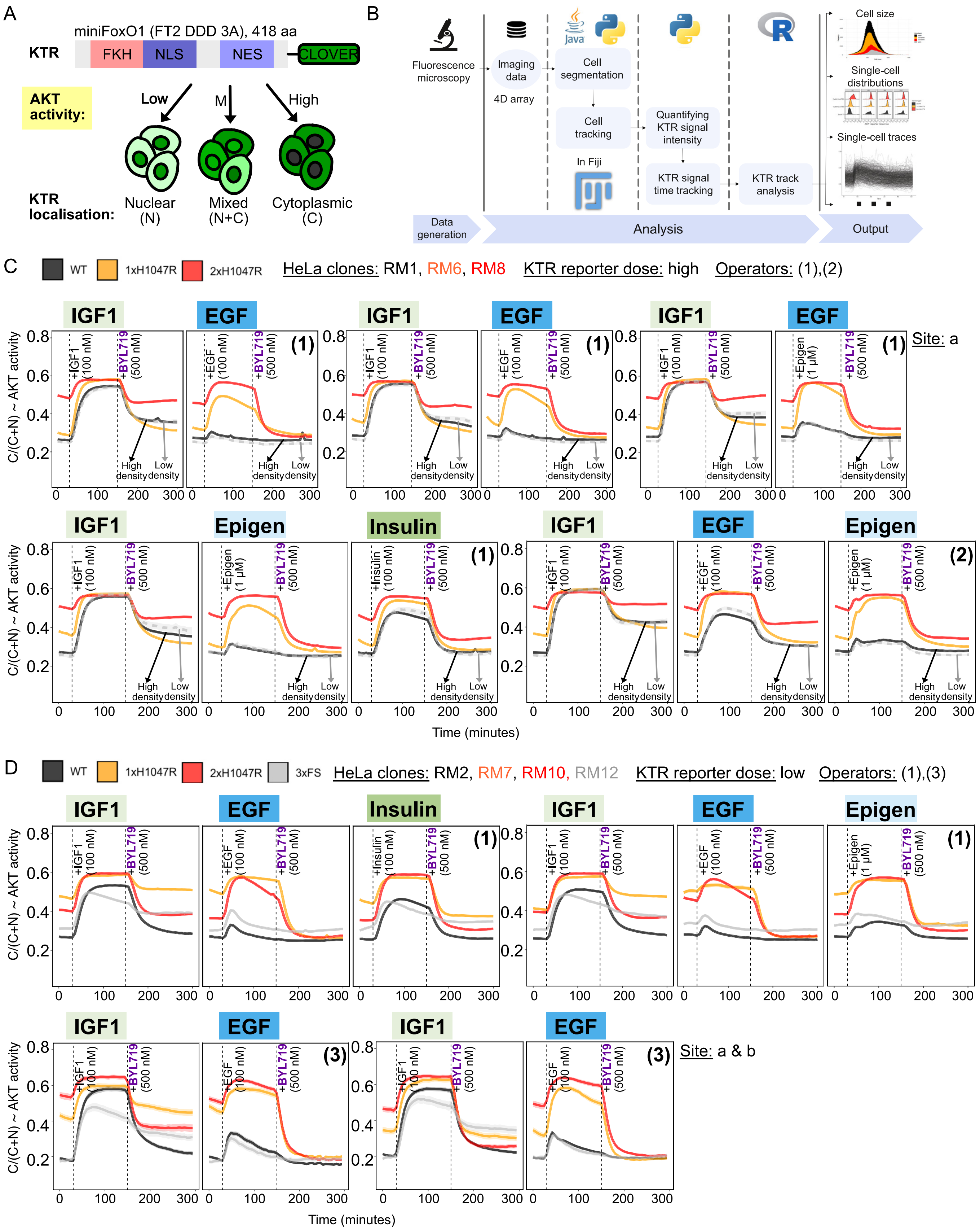
A
miniFoxO1 (FT2 DDD 3A), 418 aa
KTR
FKH
NLS
NES
CLOVER
AKT activity:
Low
M
High
KTR localisation:
Nuclear (N)
Mixed (N+C)
Cytoplasmic (C)
B
Fluorescence microscopy
Imaging data
4D array
Java
Cell segmentation
Cell tracking
In Fiji
Quantifying KTR signal intensity
KTR signal time tracking
KTR track analysis
Cell size
Single-cell distributions
Single-cell traces
Data generation
Analysis
Output
C
WT
1xH1047R
2xH1047R
HeLa clones: RM1, RM6, RM8
KTR reporter dose: high
Operators: (1),(2)
IGF1
EGF
Epigen
Insulin
+IGF1 (100 nM)
+EGF (100 nM)
+Epigen (1 µM)
+Insulin (100 nM)
+BYL719 (500 nM)
High density
Low density
(1)
(2)
Site: a
C/(C+N) ~ AKT activity
Time (minutes)
D
WT
1xH1047R
2xH1047R
3xFS
HeLa clones: RM2, RM7, RM10, RM12
KTR reporter dose: low
Operators: (1),(3)
(3)
Site: a & b
C/(C+N) ~ AKT activity
Time (minutes)

 © The Author(s)

◀ **Figure EV2. FOXO-based AKT kinase translocation reporter (KTR) setup and full set of experimental outputs.**

(**A**) Schematic of the reporter, which was expressed stably in cells using transposon-based technology, and its mechanism of action. (**B**) Overview of the computational image and KTR data analysis pipeline which has been deposited on the accompanying OSF project site (10.17605/OSF.IO/4F69N). (**C**, **D**) The data in (**C**, **D**) are from all independent experiments performed across different genotypes, HeLa clones, cell densities, KTR reporter doses, operators and experimental sites for a robust evaluation of reproducibility. Experiments with high and low KTR transposon dose are shown in (**C**) and (**D**), respectively. For each time point, the traces correspond to the mean proportion of cytoplasmic KTR signal, with shaded areas representing bootstrapped 95% confidence intervals of the mean (note that these may be too small to be seen on the figure). Although we observed operator-dependent differences in EGF-induced signaling dynamics in WT *PIK3CA* cells, the overall pattern relative to IGF1, including the blurring of the response in mutant cells, remained consistent. This technical variability in EGF responses in WT cells is likely due to their sensitivity to the pressure/rate of delivery of the stimulus through the manual fluidics system (see https://doi.org/10.17504/protocols.io.261gedjkjv47/v1).

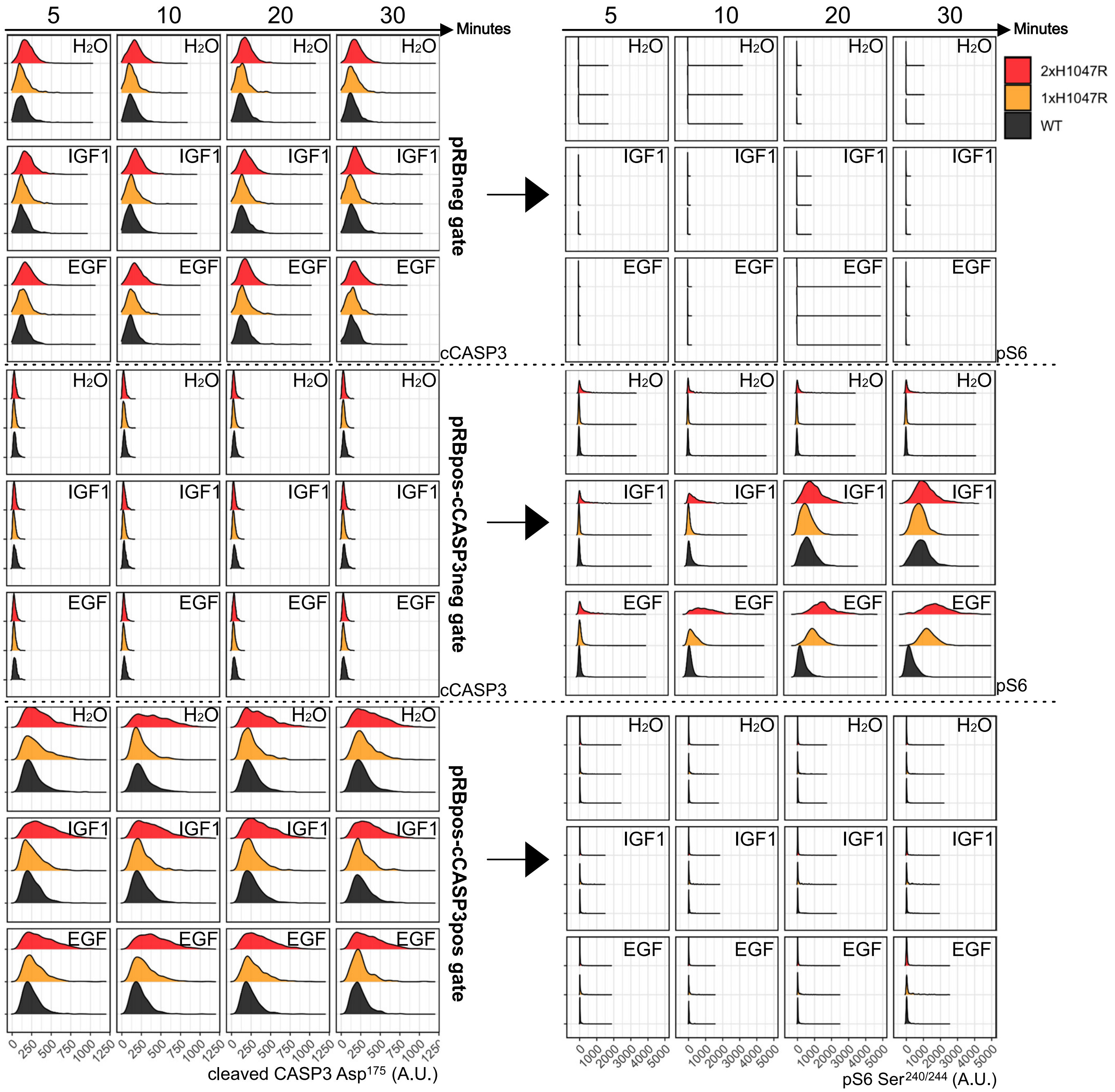

Figure EV3. Representative CyTOF data demonstrating that growth factor-induced signaling responses are observed only in cycling and non-apoptotic HeLa spheroid cells.

The plots on the left-hand side show the single-cell signal for cascade 3 (CASP3) cleaved at Asp^{175} in the different pRB gates (pRB-negative^{-} or pRB-positive^{+} at $Ser^{807/811}$). The plots on the right show the corresponding pS6 $Ser^{240/244}$ signal in each gate. The overall experimental setup is as shown in Fig. 4. The shown data are from $n = 1$ clone per genotype but are representative of four independent experiments across two independent clones per genotype.

 © The Author(s)

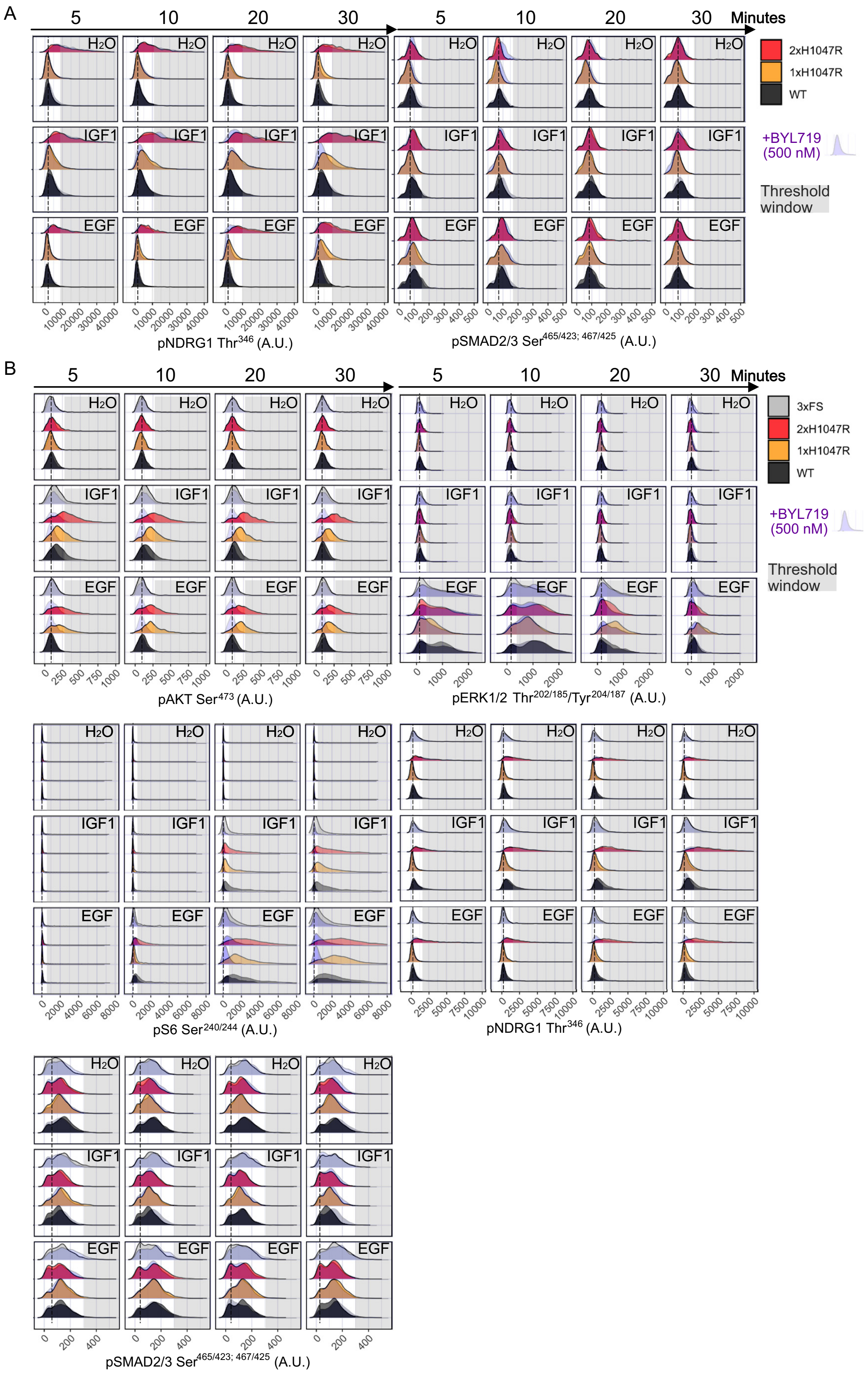
A
5
10
20
30
Minutes
H2O
IGF1
EGF
2xH1047R
1xH1047R
WT
+BYL719
(500 nM)
Threshold
window
pNDRG1 Thr346 (A.U.)
pSMAD2/3 Ser465/423; 467/425 (A.U.)
B
3xFS
pAKT Ser473 (A.U.)
pERK1/2 Thr202/185/Tyr204/187 (A.U.)
pS6 Ser240/244 (A.U.)
pNDRG1 Thr346 (A.U.)
pSMAD2/3 Ser465/423; 467/425 (A.U.)

Figure EV4. Additional CyTOF data and independent experimental replicate with independent clones.

(**A**) The pNDRG1 and pSMAD2/3 signaling responses captured as part of the dataset shown in Fig. 4. The phosphorylation of NDRG1 on Thr^{346} is a marker of mTORC2 activation (García-Martínez and Alessi, 2008). The phosphorylation of SMAD2/3 ($Ser^{465/423}$; $Ser^{467/425}$) is a marker for activated TGFβ signaling which is associated with $PIK3CA^{H1047R}$ phenotypes in human iPSCs (Madsen et al, 2021). (**B**) CyTOF data from an independent repeat of the experiment in Fig. 4, using independent CRISPR/Cas9-engineered, 3D-cultured HeLa clones, including the *PIK3CA* loss-of-function 3xFS clone as an additional control. The spheroids were serum-starved for 4 h prior to the indicated perturbations. The signaling data are from cycling, non-apoptotic cells. The stippled line indicates the position of the peak in WT spheroids treated with vehicle (H_2O). The gray shading highlights the response region not shown by WT *PIK3CA*-expressing cells in the absence of stimulation.

 © The Author(s)

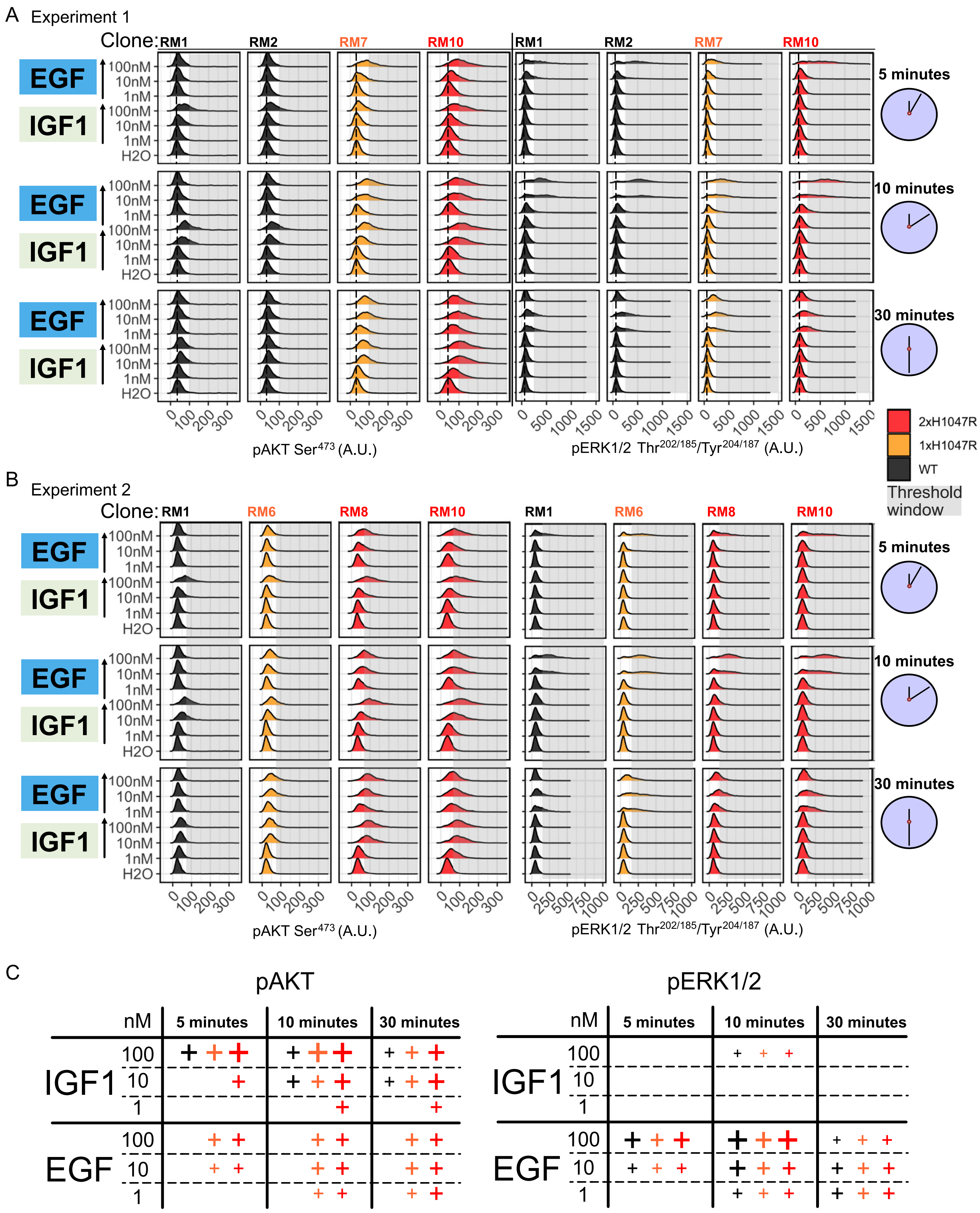
A Experiment 1
Clone: RM1 RM2 RM7 RM10 RM1 RM2 RM7 RM10
EGF
IGF1
100nM
10nM
1nM
H2O
5 minutes
10 minutes
30 minutes
0 100 200 300
pAKT Ser473 (A.U.)
0 500 1000 1500
pERK1/2 Thr202/185/Tyr204/187 (A.U.)
2xH1047R
1xH1047R
WT
Threshold window
B Experiment 2
Clone: RM1 RM6 RM8 RM10 RM1 RM6 RM8 RM10
0 250 500 750 1000
C
pAKT
pERK1/2
nM
5 minutes
10 minutes
30 minutes
IGF1
EGF
100
10
1

Figure EV5. Dose- and time-dependent IGF1 and EGF single-cell signaling responses in HeLa spheroid cells with WT or *PIK3CA*^{H1047R} (1–2 copies) expression.

(**A**, **B**) The plots in (**A**, **B**) are from two independent CyTOF datasets using independent CRISPR/Cas9-engineered, 3D-cultured HeLa clones stimulated with 1, 10 or 100 nM of IGF1 or EGF as a function of time. The spheroids were serum-starved for 4 h prior to the indicated perturbations. The signaling data are from cycling, non-apoptotic cells. The stippled line indicates the position of the peak in WT spheroids treated with vehicle (H_2O). The gray shading highlights the response region not shown by WT *PIK3CA*-expressing cells in the absence of stimulation. (**C**) Graphical summary of the key observations in the datasets in (**A**, **B**). A positive response is indicated with (+), the size of which indicates the response magnitude.

 © The Author(s)
